# Genito-Urinary and Syphilitic Diseases

**Published:** 1897-01

**Authors:** Byron H. Daggett

**Affiliations:** Buffalo, N. Y.


### GENITO-URINARY AND SYPHILITIC DISEASES.

CONDUCTED BY BYRON H. DAGGETT, M. D., Buffalo, N. Y.

### TESTING URINE.

POURING urine into a test-tube containing nitric acid, or pouring nitric acid into a test-tube containing urine usually results in a commingling of these substances without the formation of the distinct white layer, or if albumin be present in small quantity without rendering the urine turbid, since an excessive amount of acid redissolves the albumin. This way of manipulation completely impairs the test. The urine should trickle down the side of the test-tube and float over the acid, when a clear-cut white line will form; some one of the other well-known tests should also be used to confirm the finding of the nitric acid test. Albumin will not curd or precipitate by boiling in strongly acid or strongly alkaline urine, hence the boiling test should be preceded by neutralisation of this excretion.

A ready method of applying the nitric acid test was recently described in this department of the JOURNAL, which will bear repetition. The urine to be tested is drawn up into a pipette and then the acid is drawn in after it. In this way there is surface contact without commingling and a clean-cut, well-defined line of demarcation is formed.

---

### HEMAL HYDRARGYROIODIDE IN THE TREATMENT OF ADVANCED SYPHILIS.

KOBERT (*Centralblatt für Nervenheilkunde und Psychiatrie*, May, 1896; *Fortschritte der Medicin*, November 1, 1896; *New York Medical Journal*, December 5, 1896,) recommends this preparation, said to contain 13 per cent. of mercury and 28 per cent. of iodine, in combination with blood pigment.

This combination is said not to irritate the mucous surfaces and passes through the stomach without being dissolved. It not only produces the specific effects of mercury and iodine, but also exerts a mild chalybeate action. Kobert advises the following combination:

| | | |
|---|---|---|
| ℞ | Hemal hydrargyroiodide .. .................. | 150 gr. |
| | Opium .. ....................................... | 15 gr. |
| | Glycerin ointment .. ........................ | q. s. |

Mix and divide into a hundred pills. At first give one and gradually increase to four pills, three times daily.

THE SERUM TREATMENT OF SYPHILIS.

TARNOWSKY (abstract from editorial in the *New York Medical Journal*, December 5, 1896,) argues that it is most rational to use serum obtained by a process similar to that employed in the preparation of antidiphtheritic serum, since there are no positive results from employing the normal serum of animals, neither that of animals inoculated with syphilitic products, nor that of persons affected with constitutional syphilis.

The difficulty confronting him was to find an animal susceptible to syphilitic infection ; however, he believes he has found such an animal in the foal. He endeavored to syphilise two foals, by applying the virus to incisions, blistered surfaces and by subcutaneous injection. There were no external manifestations of this disease, but there were changes much like those due to syphilis found in different internal organs, in the blood-vessels and lymphatic glands, in the course of two or three months. After repeated inoculations, the blood was drawn from these animals and given hypodermatically to six patients.

After long persistence in this treatment, the disease ran its normal course. Erythema appeared in four cases, with pains in the muscles and joints, also purpura in two instances. Large doses decidedly increased the temperature.

Rekowski (*Gazeta Lekarska*) subjected the young serum-bearing animal to a course of injections of mercury salicytate for the purpose of generating an antisyphilitic serum. This serum bore traces of mercury, as shown by chemical arts. He injected this serum into patients affected with tertiary syphilis in doses of ten cubic centimeters every third day and reports astonishing results. After the third or fourth injection gummatous lesions rapidly disappeared.

Was this a roundabout way of administering albuminate of mercury ?

---

THE PRESERVATION OF URINARY SEDIMENT.

GUNPRECHT (*The Medical Examiner*, November, 1896,) gives a new method of preserving urinary sediments. He centrifugates the urine to form a compact sediment. For safety and convenience the tubes used should have bulbous extremities. If the urine contains blood the sediment, after decantation, should be washed with a saturated sublimate solution, again centrifugated, decanted and the remaining sediment washed in the centrifuge six times with sterilised water or a physiological salt solution. All this may be dispensed with if there is no blood and but little albumin present. Two to 10 per cent. solution of formal is poured over the sediment, which then remains as a nubecula. Gunprecht alleges that he has preserved sediment in this way for a year, so that casts, cells of various kinds, bacteria and the like, could be examined, stained or unstained.

---

### RADICAL CURE OF VARICOCELE.

DR. FRANCIS W. MURRAY (*Annals of Surgery*, July, 1895,) divides operations for varicocele into three classes: first, the subcutaneous; second, the open; third, the method of ablation. The best results obtained by the first method is by subcutaneous ligation of the veins with sterilised silk or catgut ligatures; this method is simple and easy of performance, and with antiseptic precautions is safe and necessitates but a short period of confinement. It is applicable only in cases of small varicocele requiring only a single ligature. It is uncertain as regards a radical cure, as we can never be sure that all the affected veins are included in the ligature, which is absolutely essential to prevent recurrence; again, there is liability of puncturing a vein, an event which may be attended with unfortunate results.

Since the introduction of antiseptic wound treatment, the preferable open operation has been revived. This operation has been modified by W. H. Bennett, who excises the dilated veins and ties their stumps together. With a patient in a standing position, he estimates the degree of elongation of the cord and decides the amount of varicocele to be removed. He makes an incision an inch and a half in length and exposes the fascia surrounding the veins. The catgut ligature is passed around this fascia, including the veins, at a point as near the testicle as thought proper and tied, the ends being left long. Above the ligature the sheath of the varicocele is freed by the finger from the surrounding tissues sufficiently long to be drawn out of the wound; the second ligature is then passed around the upper end, tied and the ends left long; the varicocele between the two ligatures is excised at not less than a quarter of an inch from the corresponding ligatures; the two stumps are then drawn together and tied with the ligature left for that purpose. The fascia being left intact, all the affected vessels are included within the ligature and the veins cannot be drawn out from their sheath, which is included in the ligature and helps strengthen the union. There is little or no pain resulting ; the patient is confined to his bed about a week and the suspensory bandage may be left off in a month. The open method performed in this way is simple, certain and without risk. Dr. Murray reports five cases of excision of the veins according to Bennett's method. All healed by primary union, and the results were thoroughly satisfactory ; the average duration of confinement was fourteen days ; four cases were traced from six months to two years after the operation and results remained very satisfactory. The doctor states it as his opinion that the Bennett operation is the one most likely to effect the radical cure of varicocele.

